# Editorial: The biological functionality of sleep

**DOI:** 10.3389/fnins.2023.1219904

**Published:** 2023-06-01

**Authors:** Fabio García-García

**Affiliations:** Departamento de Biomedicina, Instituto de Ciencias de la Salud, Universidad Veracruzana, Xalapa, Veracruz, Mexico

**Keywords:** EEG, metabolisms, sleep spindles, dementia, visual attention

Sleep is a fundamental aspect of human physiology that is crucial for maintaining physical and mental health. Despite its importance, many aspects of the relationship between sleep and brain function remain poorly understood. In this special issue on sleep function, we present a collection of articles that explore some of the most intriguing questions related to this complex relationship.

One of the most pressing questions in sleep research concerns the bidirectional association between sleep and brain atrophy in aging. As we age, our brains undergo a gradual process of degeneration that can lead to cognitive decline and dementia. Recent studies have suggested that poor sleep quality may accelerate this process by promoting brain atrophy, while conversely, sleep may help protect against brain degeneration (Kokošová et al.).

Another important aspect of sleep function is its relationship to visual attention. It is well-established that poor sleep quality can impair visual attention, but the precise mechanisms underlying this relationship are not fully understood. Recent studies have suggested that disruptions in the sleep-wake cycle may interfere with the activity of brain regions involved in visual attention, leading to deficits in this critical cognitive process (Abdolalizadeh and Nabavi).

In addition to its impact on cognitive function, sleep also plays a crucial role in regulating the activity of nuclei involved in the sleep-wake cycle. Recent studies have identified two key hormones, leptin, and adiponectin, that appear to play a critical role in this process. These hormones help to regulate the activity of nuclei involved in sleep and wakefulness, leading to more restful and restorative sleep (Ramírez-Plascencia et al.).

Finally, recent studies have identified functional differences in cerebral activation between slow wave-coupled and uncoupled sleep spindles. These findings suggest that different types of sleep spindles may play distinct roles in memory consolidation and other cognitive processes. In this issue, we present several articles that explore the functional differences between slow wave-coupled and uncoupled sleep spindles, shedding new light on the intricate relationship between sleep and brain function (Baena et al.).

In conclusion, this special issue on sleep function highlights the latest advances in our understanding of the complex interplay between sleep and brain function. We hope that these articles will inspire further research in this fascinating field and ultimately lead to new treatments and interventions for sleep-related disorders.

## Author contributions

FG-G wrote and reviewed the manuscript.

